# Molecular Imaging and Therapy of Merkel Cell Carcinoma

**DOI:** 10.3390/cancers6021020

**Published:** 2014-04-29

**Authors:** Volkan Beylergil, Jorge A. Carrasquillo

**Affiliations:** 1 Molecular and Imaging Therapy Service, Department of Radiology Box 77, Memorial Sloan-Kettering Cancer Center 1275 York Ave, New York, NY 10065, USA; E-mail:beylergv@mskcc.org; 2 Department of Radiology, Weill Cornell Medical Center, New York, NY 10065, USA

**Keywords:** Merkel cell carcinoma, FDG, PET/CT, fluorodeoxyglucose, molecular, imaging, sentinel node

## Abstract

Several molecular imaging modalities have been evaluated in the management of Merkel cell carcinoma (MCC), a rare and aggressive tumor with a high tendency to metastasize. Continuous progress in the field of molecular imaging might improve management in these patients. The authors review the current modalities and their impact on MCC in this brief review article.

## 1. Introduction

Merkel cell carcinoma (MCC) is a rare malignant tumor first described in 1972 by Toker [1]. MCC is an aggressive tumor of the skin that arises from neuroendocrine cells within the basal layer of the epidermis, affecting mainly sun-exposed areas with high tendency for metastatic disease [2]. Although the exact etiology remains unknown, viral carcinogenesis is suggested. Approximately 80% of tumors have Merkel cell polyomavirus and MCC might coexist with other skin or hematopoietic malignancies [3]. Typical sites of metastatic disease include lymph nodes, skin, lung, CNS and bone [4] and prognosis is worse than melanoma [5]. Treatment of the primary consists of surgical resection for the purpose of cure or for improved regional control. MCC is considered radiosensitive and radiation therapy plays a role in almost every clinical scenario. Radiotherapy may be used for definitive (curative) or adjuvant treatment of primary tumor, and definitive, adjuvant and prophylactic treatment of lymph node basin. For a complete discussion of the role of radiation treatment in MCC, readers are strongly encouraged to read a recent review article [6].

Given that, pre-treatment staging is an independent predictor of survival [7] nodal sampling is frequently performed to avoid the need for total neck dissection [8]. Because of significant metastatic potential and frequent reoccurrence patients often undergo computed tomography (CT scan) for evaluation of extent of disease and surveillance [9]. Nonetheless, CT cannot differentiate tumor in nodes smaller than 1 cm and detection rate of 20% for nodal disease has been described at initial staging [10]. Given the limitations of anatomical imaging modalities such as CT, the use of molecular and hybrid imaging plays an important role in the management of MCC.

## 2. Molecular Imaging and Therapy of Merkel Cell Carcinoma

### 2.1. Potential Agents

Various radiolabeled compounds that take advantage of molecular pathways that are present in neuroendocrine tumor have been evaluated in MCC [11]. MIBG is a radiolabeled analogue of guanethidine that enter cells via NET transporter and is either stored in the cytoplasm or in secretory granules. Von Moll *et al.* studied MIBG avidity of different neuroendocrine tumors and reported one case of MCC with ^131^I-MIBG uptake [12]. In another study, one of two cases of MCC was detected on ^131^I-MIBG scan [13]. More recently, ^123^I-MIBG is used rather than ^131^I-MIBG because of more favorable imaging characteristics. A case report of ^123^I-MIBG imaging of MCC has appeared [14]. Given the limited studies no role can be advocated for MIBG imaging.

**Figure 1:**
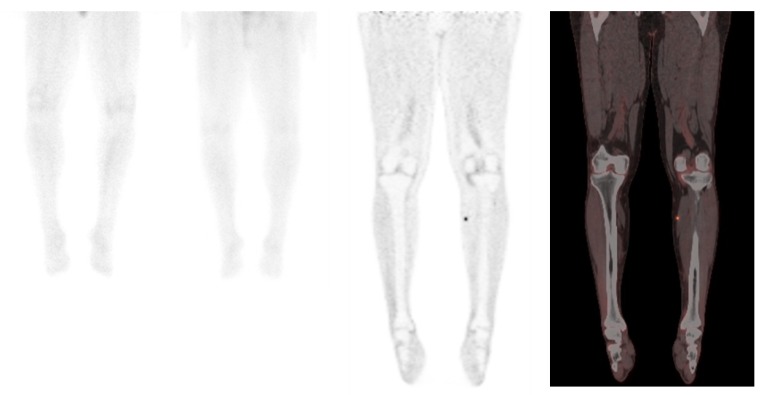
FDG PET/CT shows a focal milimetric focus not visualized on octreotide scan a day prior to PET/CT scan.

**Figure 2:**
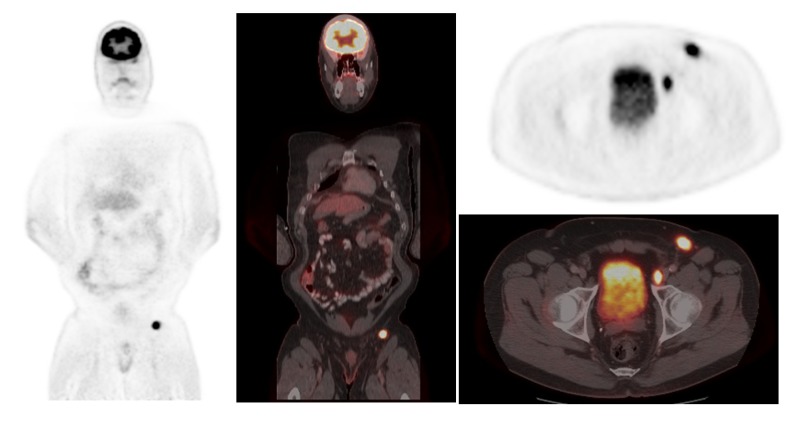
FDG PET/CT showing left inguinal node metastases in a patient with Merkel cell carcinoma.

**Figure 3:**
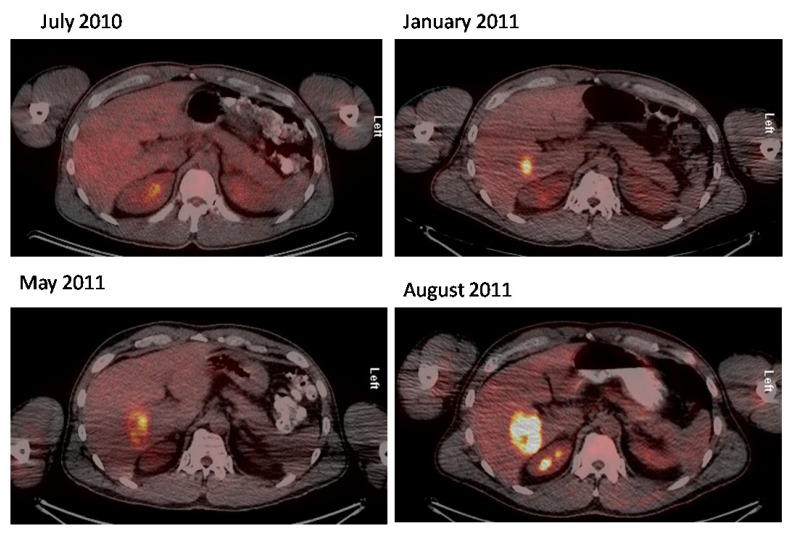
A series of axial fused PET/CT images showing gradual progress in liver metastasis from Merkel cell carcinoma.

**Figure 4:**
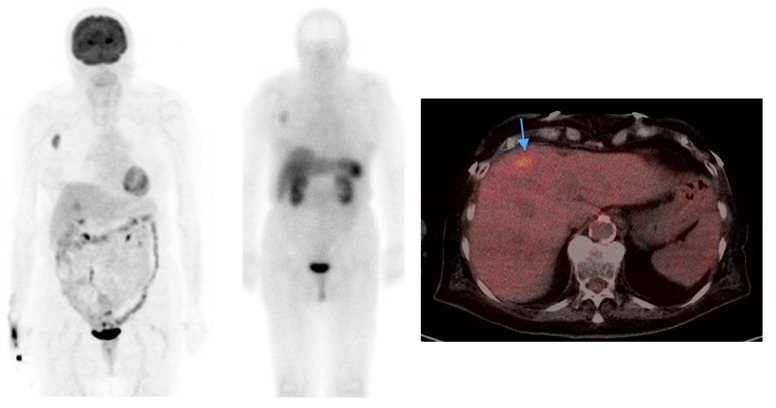
Subtle liver metastasis in a Merkel cell carcinoma patient that is not appreciable on octreotide scan. Right axillary nodal metastasis, on the other hand is visible on both studies.

A limited number of reports using somatostatin receptor scintigraphy (SRS) in MCC have appeared in the literature. The potential utility of this technique is based on the presence of somatostatin receptor type 2, with one report demonstrating presence of this receptor by RT-PCR in nine of 10 patients examined [15]. Kwekkeboom studied five patients with CT and SRS and detected uptake in four of five cases with both, but additional lesions were positive with SRS compared to CT [16]. In contrast, Durani *et al.* studied 11 patients with SRS and showed positive uptake in four of seven patients whereas false negative or false positive were seen in five of 11 [17]. In a larger series Guitera-Rovel evaluated 20 patients with Merkel cell tumor with SRS [18]. Their sensitivity was 78% and specificity was 95%. Overall four of the five primary and six of eight lymph node metastasis, two of three thoracic metastases and zero out of two liver metastases and none of the metastatic skin lesion in two patients were visualized [18]. In a group of six patients with MCC SRS detected all three patients with active disease [19]. In these reports SRS typically did [20] not add additional information beyond conventional imaging to recommend it routinely [7,18]. A head to head comparison of SRS to FDG in a group of nine patients showed that FDG outperformed SRS with no SRS positive FDG negative lesions, implying SRS is suboptimal for imaging MCC [21] (Figure 1, Figure 2, Figure 3 and Figure 4). Nonetheless a potential role for SRS is as a theranostic reagent for selection of patients who may be candidates for ^90^Y/^177^Lu somatostatin analog receptor directed therapy [22,23]. 

The use of ^68^Ga labeled somatostatin analogs such as DOTATOC, DOTATATE and DOTANOC has been shown to be far superior to that of ^111^In pentetretotide SRS. Preliminary case reports have indicated that ^67^Ga-DOTATATE can localize well in MCC, with one case showing better localization than FDG [22,24,25]. In order to determine if there is any role for these new ^68^Ga PET tracers additional studies need to be performed evaluating their utility in MCC.

Fluorodopa is an ^18^F labeled amino acid analog that enter the cell through LAT transporters and has been shown useful in a variety of neuroendocrine tumors [11]. There have been preliminary reports of ^18^F-DOPA in small number of MCC [19,26]. Although the limited number of patients prevents general conclusion, it appears that it has less contrast and sensitivity than FDG for MCC.

### 2.2. ^18^F-FDG PET/CT in Merkel Cell Carcinoma

Broader experience is available with FDG (Table 1). Overall FDG has had good sensitivity for detecting nodal or metastatic disease. The intensity of uptake is high with mean SUV_max_ values ranging from ~4 to 13.6 [27,28,29] The sensitivity for tumor detection is high, ranging from 79%–94% with very high specificity, >96% (Table 1). Interestingly the Ki-67 index is elevated, with a mean value of 50% which in other neuroendocrine tumor has been associated with increased FDG uptake [20,27]. In one report comprising 21 patients, FDG results changed staging in 33% of the patients with MCC and altered management in 43% of the patients [28]. Hawryluk *et al.* retrospectively reviewed 270 FDG PET/CT studies performed in 97 patients and concluded that FDG resulted in upstaging in 16% of patients [30] similarly a retrospective single center study reported changes in tumor status in 20% of patients [31]. Recently, Siva *et al.* showed that FDG played a very important role in the risk stratification and management of MCC patients [32]. In their large series involving prospective analysis of 102 patients, they observed that PET changed the management plan in 37% of patients which was in parallel with prior studies in smaller series and somewhat more than 15% reported by Ibrahim *et al.* [31]. They also showed that presence of nodal disease on PET and the presence of any metabolically active disease on PET were associated with decreased overall and progression free survival. Overall, MCC lesions were highly FDG avid in this series, similar to previous reports with lower number of patients. They reported a mean SUV max of 10.3, however there was no association between SUV values and prognosis.

**Table 1:** Sensitivity and specificity of FDG PET/CT *vs.* somatostatin receptor scintigraphy.

Author (number of patients)	SRS sensitivity	SRS specificity	FDG sensitivity	FDG specificity
[18] *n* = 20	78%	96%	NA	NA
[28] *n* = 21	NA	NA	94%	100%
[27] *n* = 11	NA	NA	92%	100%
[19] *n* = 16	NA	NA	85.7%	96.2%

A recent meta-analysis on diagnostic performance of FDG PET/CT involving ten studies with a total number of 329 patients, reported sensitivity of 90% and specificity of 98% [33]. A prospective trial sponsored by Trans-Tasman Radiation Oncology Group (TROG) is currently accruing patients and will help further clarify the role of PET/CT in patients with MCC (NCT01013779).

There are no head to head comparisons of functional imaging modalities nor are there prospective comparisons of functional and anatomical imaging. Colgan *et al.* reported in a retrospective study that FDG is far more sensitive than CT in assessing nodal involvement in patients with MCC [34]. Although there are no guidelines on scan coverage, head to toe imaging appears to be appropriate to visualize possible metastases in the distal parts of the skeleton and cranium [35].

In summary given the high incidence of regional and metastatic disease and their effect on prognosis, and the high sensitivity of FDG for MCC it appears to have a role in the evaluation of these patients when regional and metastatic disease is suspected, although well designed prospective studies have not been performed.

## 3. Sentinel Node Biopsy in Merkel Cell Carcinoma

Sentinel lymph node is the first node or group of nodes draining a tumoral mass. SLN concept is not new and successful localization of sentinel node with a gamma probe dates back to 1993 [36]. SLN has been widely used for various malignancies including breast cancer, melanoma, gynecologic and penile cancers.

**Figure 5:**
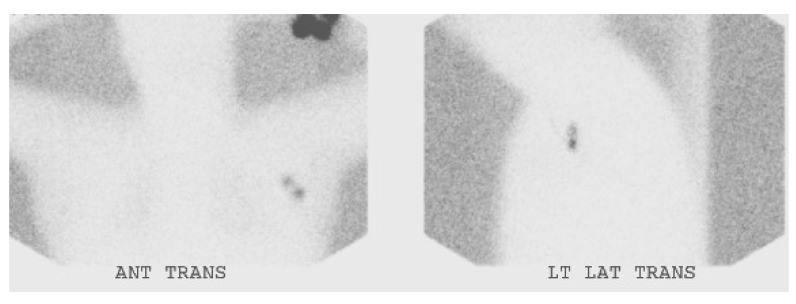
Sentinel node scintigraphy in a patient with MCC of the left forearm.

The role of sentinel node biopsy (SLNB) in MCC is not as well established as in early stage breast cancer or melanoma, partially owing to lack of prospective studies due to the fact that MCC is a very rare skin malignancy. It is still unclear whether a positive SLNB in MCC has therapeutic and prognostic implications. Gupta *et al.* reported on their institutional experience combined with a meta-analysis of the existing cases in the literature [10]. They concluded that the recurrence rate was three times higher in the SLNB positive patients compared with SLNB negative group. SLNB upstaged about one third of patients who would have been categorized as stage I based on clinical criteria. They recommended routine use of SLNB for MCC patients. Largest single center study in the literature performed by investigators from the Memorial Sloan-Kettering Cancer Center reported 29% SLNB positivity in clinically node negative patients. In their series comprising 153 patients, there was no statistically significant association between SLNB status and recurrence and survival [37]. Interestingly, 26% of patients with primary tumors measuring 1 cm or less had positive SLNB. They concluded that presence of lymphovascular invasion is a strong predictor of positive SLNB. On the other hand, Schwartz *et al.* identified clinical size, greatest histologic dimension, tumor thickness and mitotic rate as predictors of SLNB positivity [38]. In a group of 93 patients, 23.8% of patients had SLNB positivity despite having a small clinical primary tumor measuring 1 cm or less. Therefore, both groups have recommended routine SLNB for MCC patients without clinical evidence of lymph node metastases. Although Stokes *et al.* recommended SLNB only for patients with tumors 1 cm or greater [39], based on above reviews tumor diameter is not a reliable criteria to preclude SLNB. (Figure 5) Sattler *et al.* demonstrated increased overall survival benefit of 211 months in patients who underwent SLNB compared to 72 months without SLNB procedure. Kachare *et al.* [40] recently analyzed 1193 patients in the Surveillance, Epidemiology, and End Results (SEER) registry and found that SLNB negativity was associated with improved MCC-specific survival (84.5% *vs.* 64.6%). However, better outcome in the SLNB positive group might be confounded by different management strategies in this group. The value of SLNB might also differ according to body region. For example, Fritsch *et al.* reported an analysis of 721 patients with head and neck MCC in the SEER database where they could not show a survival advantage in SLNB negative group [41].

False negative rate of SLNB in MCC tend to be higher than other cancers. Howle *et al.* reported a false negative rate of 20% and more recently Shibayama *et al.* reported a false negative rate of 12.9% in a group of 403 patients [42]. However, false negative rate is much lower for melanoma [43].

Although controversies exist regarding the impact of SLNB in the management of MCC, NCCN guidelines recommend SLN biopsy for all clinically N0 patients before wide surgical excision [44]. Because of resolution issues FDG is less sensitive for picking up small nodal disease than SLN [30].

The combination of blue dye, gamma probe and lymphoscintigraphy is the most accurate approach for successful localization. The rate of non-visualization is low in the literature. Schwartz *et al.* reported non-visualization in 4% of cases [38]. However, Maza *et al.* and Fields *et al.* were able to identify at least one sentinel node in all of their patients [8,37]. New techniques such as SPECT/CT and intraoperative cameras might further increase the sentinel node detection rate and help in complicated cases where primary tumor is close to the drainage site. Recently, there has been interest in combining new radiopharmaceuticals such as Zr-89 nanoalbumin with PET/CT [45].

## 4. Conclusions

In summary, F-18 FDG PET/CT stands out as the best molecular imaging method for Merkel cell carcinoma. Well designed prospective studies are needed to elucidate the role of SLNB procedure in MCC.
